# Pharmacokinetic Interactions between Canagliflozin and Sorafenib or Lenvatinib in Rats

**DOI:** 10.3390/molecules27175419

**Published:** 2022-08-24

**Authors:** Yanjun Cui, Ying Li, Caihui Guo, Yajing Li, Yinling Ma, Zhanjun Dong

**Affiliations:** 1 National Clinical Drug Monitoring Center, Department of Pharmacy, Hebei Province General Center, Shijiazhuang 050051, China; 2 Department of Pharmacy, Hebei General Hospital, Shijiazhuang 050017, China

**Keywords:** pharmacokinetics, sorafenib, lenvatinib, canagliflozin, drug–drug interaction

## Abstract

Hepatocellular carcinoma (HCC) and type 2 diabetes mellitus (T2DM) are common clinical conditions, and T2DM is an independent risk factor for HCC. Sorafenib and lenvatinib, two multi-targeted tyrosine kinase inhibitors, are first-line therapies for advanced HCC, while canagliflozin, a sodium-glucose co-transporter 2 inhibitor, is widely used in the treatment of T2DM. Here, we developed an ultra-performance liquid chromatography-tandem mass spectrometry method for the simultaneous determination of canagliflozin, sorafenib, and lenvatinib, and investigated the pharmacokinetic drug interactions between canagliflozin and sorafenib or lenvatinib in rats. The animals were randomly divided into five groups. Groups I–III were gavage administrated with sorafenib, lenvatinib, and canagliflozin, respectively. Group IV received sorafenib and canagliflozin; while Group V received lenvatinib and canagliflozin. The area under the plasma concentration-time curves (AUC) and maximum plasma concentrations (C_max_) of canagliflozin increased by 37.6% and 32.8%, respectively, while the apparent volume of distribution (V_z/F_) and apparent clearance (CL_z/F_) of canagliflozin significantly decreased (30.6% and 28.6%, respectively) in the presence of sorafenib. Canagliflozin caused a significant increase in AUC and C_max_ of lenvatinib by 28.9% and 36.2%, respectively, and a significant decrease in V_z/F_ and CL_z/F_ of lenvatinib by 52.9% and 22.7%, respectively. In conclusion, drug interactions exist between canagliflozin and sorafenib or lenvatinib, and these findings provide a reference for the use of these drugs in patients with HCC and T2DM.

## 1. Introduction

Hepatocellular carcinoma (HCC) is one of the most common malignant tumors worldwide with a poor prognosis, high recurrence rates, and high mortality rates. Diabetes mellitus is a group of metabolic diseases with a high incidence rate and prone to a variety of complications, which brings about a huge economic burden to society [1,2]. There is growing evidence that type 2 diabetes mellitus (T2DM) is an independent risk factor for HCC, and it can increase the risk of HCC [3,4,5]. Therefore, the combined use of drugs to treat HCC and T2DM is very common in clinical settings.

**Figure 1:**
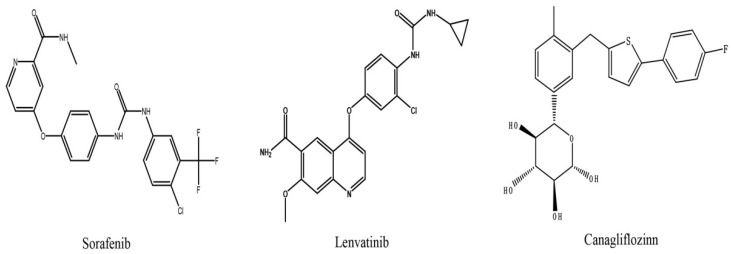
The structure of sorafenib, lenvatinib, and canagliflozin.

Sorafenib (Figure 1), a multi-kinase inhibitor with poor solubility and high membrane permeability, can inhibit tumor angiogenesis and tumor cell proliferation. It was the first targeted drug to be approved as a first-line systemic therapy for patients with advanced HCC [6,7,8]. Sorafenib reaches peak blood concentrations approximately 3 h after oral administration, with a plasma protein binding rate of 99.5%. The metabolism of sorafenib is associated with the phase I metabolic enzyme CYP3A4 and the phase II metabolic enzyme uridine diphosphate-glucuronosyltransferase (UGT) 1A9, and it is metabolized by CYP3A4 to the active metabolite sorafenib N-oxide and by UGT1A9 to the inactive metabolite sorafenib glucuronide [9,10,11]. More importantly, studies have shown that sorafenib can inhibit UGT1A1 and UGT1A9 activity and increase the exposure of irinotecan and its active metabolite SN-38 by inhibiting UGT1A1 [9,12]. Sorafenib is a substrate for multiple transporters. For instance, organic cation transporter (OCT) 1 and organic anion transporting polypeptide (OATP) 1B1/3 can mediate the hepatic uptake of sorafenib, and sorafenib can be pumped out by the efflux transporters P-glycoprotein (P-gp), breast cancer resistance protein (BCRP), and multidrug resistance-associated protein (MRP) [13,14].

Lenvatinb (Figure 1) is another first-line systemic treatment drug for advanced HCC due to its non-inferiority to sorafenib in terms of overall survival [15]. It is an oral, multi-target tyrosine kinase inhibitor that can inhibit vascular endothelial growth factor receptor (VEGFR) 1–3, fibroblast growth factor receptor (FGFR) 1–4, platelet-derived growth factor receptor (PDGFR) α, stem cell factor receptor (KIT), and rearranged during transfection (RET) [16]. Lenvatinb has low solubility, and it is rapidly absorbed after oral administration, with peak blood concentrations between 1 and 4 h. A high-fat diet slows the absorption of lenvatinib, but has no significant effect on systemic exposure [17]. The plasma protein binding rate of lenvatinib is approximately 98%, and it is mainly bound to albumin. Lenvatinib is metabolized by multiple pathways, including the CYP450 pathway and non-CYP450 pathways such as those involving aldehyde oxidase. In vitro studies have shown that the CYP3A4 pathway is critical for the oxidative metabolism of lenvatinib, and a recent study has demonstrated that CYP1A1 plays an important role in the metabolism of lenvatinib [18,19]. Lenvatinib is extensively metabolized before excretion, and its half-life is 28 h. Interestingly, body weight can affect the pharmacokinetics of lenvatinib, so patients with HCC should be given different doses depending on their body weight [20,21]. In addition, lenvatinib is a substrate for P-gp and BCRP. Single-dose rifampicin, an inhibitor of P-gp, can cause an increase in systemic exposure of lenvatinib [22], and ABCB1 gene (encodes P-gp) polymorphisms can affect the clearance and systemic exposure of lenvatinib [23].

Canagliflozin (Figure 1), the first approved sodium–glucose co-transporter 2 inhibitor (SGLT2i), is a novel type of glucose-lowering drug that reduces the reabsorption of glucose by the kidneys, thereby enhancing the excretion of glucose and lowering blood sugar levels [24]. Canagliflozin is a Biopharmaceutics Classification System class IV drug with low solubility and permeability. Canagliflozin is rapidly absorbed after oral administration, and the area under the plasma concentration–time curves (AUC) and maximum plasma concentrations (C_max_) increased in a dose-dependent manner within a range of 50–1600 mg. It has a high protein binding rate of 99%. Canagliflozin is mainly glucuronidated by UGT1A9 and UGT2B4, with a small amount metabolized by CYP3A4 [25], and it is a substrate of BCRP, as well as a substrate and weak inhibitor of P-gp and MRP2 [26]. Canagliflozin is excreted by both hepatic and renal channels, with approximately 60% ending up in the feces and 33% in the urine [27,28].

Recently growing evidence has indicated that SGLT2i exhibits potential anti-tumor activity in specific types of cancer, which correlates with the presence or overexpression of SGLT2 on the surface of some specific cancer cells [29,30]. Studies have showed that SGLT2 is expressed in liver cancer cells, and canagliflozin can inhibit the proliferation of liver cancer cells by blocking glucose uptake [31,32], while another study has reported that canagliflozin can inhibit the proliferation of liver cancer cells independently of SGLT2 inhibition, possibly by blocking the glucose influx-induced β-catenin signaling pathway [33]. Therefore, canagliflozin is a promising drug in patients with HCC and T2DM. Sorafenib and lenvatinib, which inhibit angiogenesis and suppress tumor proliferation, are first-line therapies for advanced HCC, and their common adverse effect is hypertension [7,15]. Canagliflozin not only lowers blood sugar levels, but also lowers blood pressure and body weight [24]. Thus, canagliflozin combined with sorafenib or lenvatinib are very common and excellent combinations in clinical practice. However, canagliflozin and sorafenib are metabolized by UGT1A9; while sorafenib is a potent inhibitor of UGT1A9. On the other hand, they are both substrates of the efflux transporters P-gp, BCRP, and MRP, and canagliflozin has a weak inhibitory effect on P-gp and MRP. Thus, pharmacokinetic drug interactions between the two drugs may exist, which may be mediated by metabolizing enzymes and/or transporters. Similarly, lenvatinib is also a substrate for P-gp and BCRP, and canagliflozin and lenvatinib have the potential to compete for the same transporter protein, which may alter the pharmacokinetics profiles of one or both drugs.

HCC and T2DM are diseases with high incidence rates worldwide, and T2DM is a risk factor for HCC. Thus, it is possible to simultaneously prescribe canagliflozin and sorafenib or lenvatinib. In this study, we developed an ultra-performance liquid chromatography-tandem mass spectrometry (UPLC–MS/MS) method for the simultaneous determination of canagliflozin, sorafenib, and lenvatinib concentrations, and investigated the pharmacokinetic interactions between canagliflozin and sorafenib or lenvatinib in rats.

## 2. Results

### 2.1. Method Development and Optimization

An UPLC-MS/MS bioanalytical method was developed for the simultaneous determination of drug concentrations for sorafenib, lenvatinib, and canagliflozin, and the method was optimized to meet the requirements for the simultaneous determination of the three drugs in rat plasma. The aqueous phase (A), after adding 5 mM ammonium acetate and 0.1% formic acid, improved the response of canagliflozin and the peak shape of the analytes, and the organic phase (B), which was acetonitrile, showed a stronger elution capacity than methanol. Signal acquisition of the analytes was performed in positive ion and multi-response monitoring mode. [M + NH4]+ was used as the parent ion for canagliflozin because it was stable and exhibited a great response. The reaction monitoring transitions were as follows: sorafenib, *m*/*z* 465.2→270.2; lenvatinib, *m*/*z* 427.0→370.0; and canagliflozin, 462.1→191.3. The use of stable isotope-labeled IS could maximize the elimination of ionization differences and reduce matrix effects. Liquid–liquid extraction was cleaner than protein precipitation, with less matrix effect, which was suitable for the high-throughput determination of samples [34]. The extraction efficiency of the different extractants was compared, and the results showed that sorafenib was extracted well in methyl tert-butyl ether, while lenvatinib was extracted well in ethyl acetate. To obtain a better extraction recovery, a mixture of methyl tert-butyl ether and ethyl acetate extractant was used.

### 2.2. Method Validation

**Figure 2:**
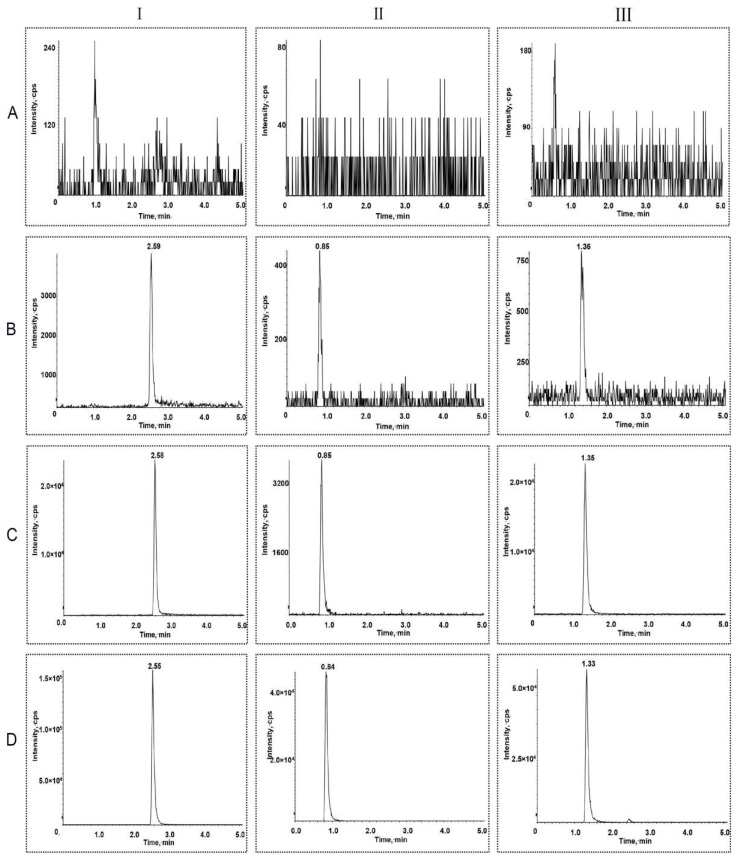
Representative chromatograms of the sorafenib (I), lenvatinib (II), and canagliflozin (III) in rat plasma samples. (**A**) Rat blank plasma samples; (**B**) rat blank plasma samples spiked with analytes at the lower limit of quantification level; (**C**) rat plasma samples after oral administration of analytes; and (**D**) internal standards in the lower limit of quantification samples.

Representative chromatograms of sorafenib, lenvatinib, and canagliflozin under different conditions are shown in Figure 2, and the results showed that endogenous substances in rat plasma did not interfere with the determination of the three analytes. The linearity of sorafenib, lenvatinib, and canagliflozin was determined over the concentration ranges of 5–5000 ng/mL, 0.2–1000 ng/mL, and 5–3000 ng/mL, respectively. Representative linear regression equations for the calibration curve were established as follows:

Y = 0.00389 X + 0.00535  Sorafenib

Y = 0.0152 X − 0.000304  Lenvatinib

Y = 0.0024 X − 0.00295  Canagliflozin

The correlation coefficients were greater than 0.999, indicating good linearity. The intra-batch and inter-batch precision and accuracy for three analytes were evaluated at LLOQ and three QC levels (Table 1). The intra-batch and inter-batch precision of the three analytes were within 3.75–9.54%, and the intra-batch and inter-batch accuracy for the three analytes were within −4.56–9.73%. Three concentrations of the QC samples were used to assess the matrix effect and extraction recovery, and the results are summarized in Table 2. The results indicated that the determination of the analytes was not affected by the matrix, and the extraction recovery of the three analytes was high. The stability of the QC samples was examined under different conditions, including at room temperature for 4 h, in an autosampler after processing for 12 h, at −80 °C for 30 days, and after three cycles of freezing (−80 °C) and thawing (room temperature), and the results are summarized in Table 3. The results showed that sorafenib, lenvatinib, and canagliflozin were stable during the different processing and storage conditions.

### 2.3. Pharmacokinetic Interactions between Sorafenib and Canagliflozin

#### 2.3.1. The Effect of Canagliflozin on the Pharmacokinetics of Sorafenib

**Figure 3:**
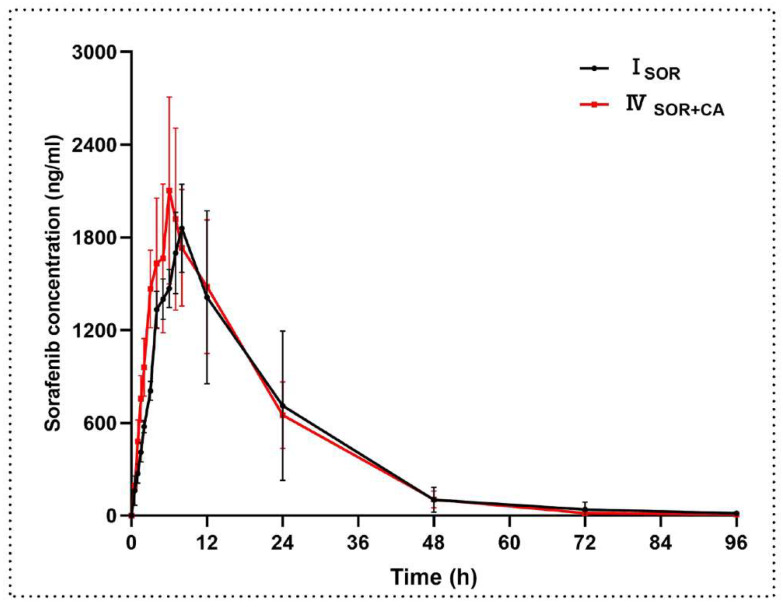
The mean plasma concentration–time curves of sorafenib after administration. Each data point represents the mean ± standard deviation (n = 6). I_SOR_, sorafenib (100 mg/kg); IV_SOR+CA_, sorafenib (100 mg/kg) oral co-administered with canagliflozin (10 mg/kg).

The mean plasma concentration–time curves of sorafenib after administration of sorafenib (100 mg/kg) or co-administration of sorafenib (100 mg/kg) and canagliflozin (10 mg/kg) are shown in Figure 3. The results showed that the main pharmacokinetic parameters of sorafenib were similar and comparable when combined with canagliflozin compared to sorafenib alone. The specific pharmacokinetic parameters are shown in Table 4.

#### 2.3.2. The Effect of Sorafenib on the Pharmacokinetics of Canagliflozin

**Figure 4:**
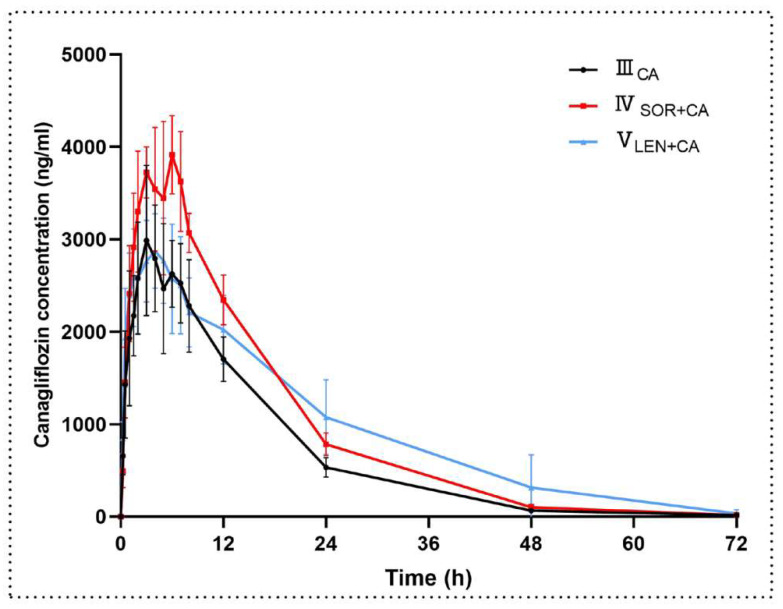
The mean plasma concentration–time curves of canagliflozin after oral administration. Each data point represents the mean ± standard deviation (n = 6). III_CA_, canagliflozin (10 mg/kg); IV_SOR+CA_, canagliflozin (10 mg/kg) oral co-administered with sorafenib (100 mg/kg); V_LEN+CA_, canagliflozin (10 mg/kg) oral co-administered with lenvatinib (1.2 mg/kg).

Figure 4 shows the mean plasma concentration–time curves after administration of canagliflozin, and the main pharmacokinetic parameters are summarized in Table 5. The results showed that the AUC_last_ and AUC_inf_ for canagliflozin increased by 37.7% and 37.6%, respectively, when canagliflozin was co-administered with sorafenib compared to canagliflozin alone. The C_max_ of canagliflozin increased by 32.8% in the presence of sorafenib, while the V_z/F_ (30.6%) and CL_z/F_ (28.6%) of canagliflozin decreased in the presence of sorafenib. However, the other pharmacokinetic parameters, including t_1/2_, did not show significant changes between the two groups.

### 2.4. Pharmacokinetic Interactions between Lenvatinib and Canagliflozin

#### 2.4.1. The Effect of Canagliflozin on the Pharmacokinetics of Lenvatinib

**Figure 5:**
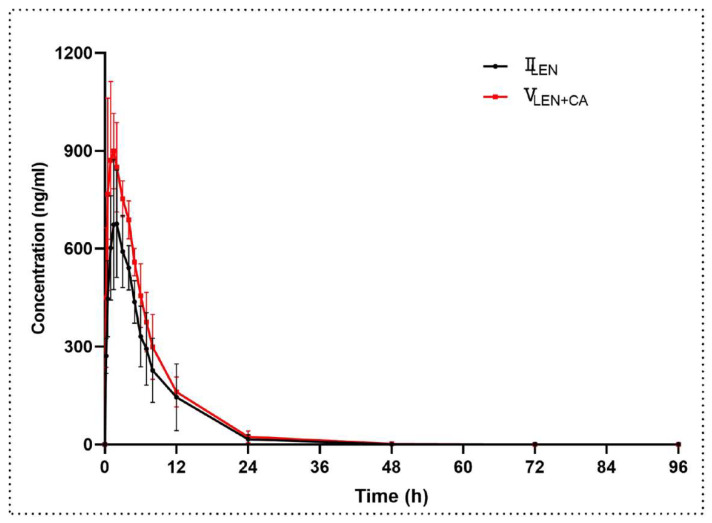
The mean plasma concentration–time curves of lenvatinib after oral administration. Each data point represents the mean ± standard deviation (n = 6). II_LEN_, lenvatinib (1.2 mg/kg); V_LEN+CA_, lenvatinib (1.2 mg/kg) oral co-administered with canagliflozin (10 mg/kg).

The mean plasma concentration–time curves of lenvatinib after oral administration of lenvatinib (1.2 mg/kg) or co-administration of lenvatinib (1.2 mg/kg) and canagliflozin (10 mg/kg) are shown in Figure 5, and the main pharmacokinetic parameters are tabulated in Table 6. The systemic exposure of lenvatinib increased when canagliflozin was co-administered with lenvatinib. In the presence of canagliflozin, the C_max_ of lenvatinib increased by 36.2% compared to lenvatinib alone. At the same time, the AUC_last_ and AUC_inf_ of lenvatinib were significantly greater by 28.9%. The t_1/2_ and V_z/F_ of lenvatinib decreased by 38% and 52.9%, respectively; and CL_z/F_ was significantly slower by 22.7% than those without canagliflozin.

#### 2.4.2. The Effect of Lenvatinib on the Pharmacokinetics of Canagliflozin

The mean plasma concentration–time curves and the main pharmacokinetic parameters of canagliflozin after oral administration of canagliflozin or co-administration of canagliflozin and lenvatinib are shown in Figure 4 and Table 5, respectively. Compared to the control group, the V_z/F_ and CL_z/F_ of canagliflozin decreased when canagliflozin was co-administered with lenvatinib. In addition, the AUC was elevated but the change was not statistically significant. The other main pharmacokinetic parameters of canagliflozin did not show significant changes.

## 3. Discussion

The combination of medications is a common approach in clinical practice. On the one hand, the combination medication can play a synergistic role in reducing the dose and toxicity or decreasing the drug resistance. On the other hand, the co-morbidity of multiple diseases and the presence of disease complications make the combination of drugs an inevitable choice. However, combination drugs may also cause drug interactions that pose unknown risks. Sorafenib and lenvatinib are the first-line drugs for the systemic treatment of advanced HCC, and canagliflozin is a novel SGLT2i for the treatment of T2DM that can also potentially inhibit the proliferation of liver cancer cells. Canagliflozin is often co-prescribed with sorafenib or lenvatinib in patients with advanced HCC and T2DM. Thus, it is important to evaluate drug interactions between canagliflozin and sorafenib or lenvatinib.

To our best knowledge, there is no method for the simultaneous determination of canagliflozin, sorafenib, and lenvatinib. In this study, a method was developed for the simultaneous determination of sorafenib, lenvatinib, and canagliflozin concentrations in rat plasma, and applied to pharmacokinetic interaction studies between canagliflozin and sorafenib or lenvatinib. This method requires only a small volume of plasma sample (50 μL) and is suitable for pharmacokinetic analysis of small volumes of animals. Liquid–liquid extraction with mixed extractants, which shows good extraction recovery and low matrix effect, enables accurate quantification. Furthermore, the method has a short analysis time and a wide range of calibration curves, which are suitable for the determination of high-throughput samples.

The dose of 100 mg/kg for sorafenib administration was selected after consulting previous studies [35,36], and the doses of 1.2 mg/kg for lenvatinib and 10 mg/kg for canagliflozin were the chosen reference for the conversion of human doses to animal doses for clinical applications [37].

Drug exposure in vivo is closely associated with drug efficacy and adverse drug reactions. Studies have shown that the plasma concentration of sorafenib is closely related to its main adverse effects, including diarrhea, fatigue, hepatic impairment, hand–foot skin reaction, and hypertension [38,39]. Similarly, the plasma drug concentration and systemic exposure of lenvatinib were associated with its efficacy and adverse effects. For instance, the incidence of hypertension and anorexia was higher in patients with higher trough concentrations of drugs [21,40,41]. More importantly, the excretion of urinary glucose in patients treated with canagliflozin was increased in concentration and dose-dependent manner [26,42]. Therefore, changes in the concentrations of drugs, when combined with certain drugs, can lead to reducing or enhancing drug efficacy or causing adverse effects.

In this study, pharmacokinetic interactions between canagliflozin and sorafenib were investigated in rats. The main pharmacokinetic parameters of sorafenib were not altered when sorafenib was co-administered with canagliflozin. Sorafenib is a substrate for efflux transporters, such as P-gp, BCRP, and MRP, and thus, transporter-mediated drug interactions may occur when the substrates, inducers, or inhibitors of transporters are applied simultaneously. On the one hand, some studies have shown that inhibitors, such as verapamil and baicalin [43,44] can significantly increase sorafenib exposure by inhibiting P-gp. On the other hand, 5,7-dimethoxyflavone, an inhibitor of BCRP, can elevate the AUC of sorafenib, possibly by inhibiting BCRP-mediated sorafenib efflux [45]. Canagliflozin is a substrate of BCRP, P-gp, MRP2, and a weak inhibitor of P-gp and MRP2. However, canagliflozin had only a slight effect on sorafenib, and thus, did not have a clinically relevant effect.

The systemic exposure of canagliflozin was significantly increased in the presence of sorafenib. The C_max_, AUC_last_, and AUC_inf_ of canagliflozin were significantly elevated, while the V_z/F_ and CL_z/F_ were significantly decreased. There are several possible reasons for the apparent discrepancy. First, sorafenib is primarily metabolized by the phase II metabolic enzyme UGT1A9, and is an inhibitor of UGT1A9 [9,10], whereas canagliflozin is mainly metabolized by UGT1A9 and UGT2B4. Therefore, sorafenib can directly inhibit UGT1A9, thereby blocking the metabolism of canagliflozin and increasing its exposure in vivo. In addition, sorafenib can also inhibit the metabolism of canagliflozin by competing with canagliflozin for the same metabolic enzymes, leading to an increase in its blood concentration. According to a study by Karbowniket al. [46], the exposure of tapentadol, which is mainly metabolized by UGT1A9 and UGT2B7, significantly increased when it was co-administered with sorafenib, indicating that UGT can mediate the interactions between the two drugs, which supports our conjecture. Second, sorafenib is not only a substrate for P-gp, BCRP, and MRP, but also an inhibitor of P-gp, while canagliflozin is a substrate for P-gp, BCRP, and MRP. As such, there may be transporter-mediated drug interactions between the two drugs.

Lenvatinib is another tyrosine kinase inhibitor that has been approved for the first-line treatment of advanced HCC. This study also investigated the drug interactions between lenvatinib and canagliflozin in vivo. The pharmacokinetic results showed that canagliflozin increased the systemic exposure and decreased the clearance and V_z/F_ of lenvatinib, while lenvatinib slowed the clearance and decreased the V_z/F_ of canagliflozin. P-gp and BCRP, two efflux transporters, are widely distributed in the body. P-gp and BCRP are expressed by epithelial cells of the intestine, and can reduce the absorption and decrease the bioavailability of substrates; P-gp and BCRP are expressed by liver cells and can increase the hepatic excretion of drugs [47,48,49,50]. Lenvatinib and canagliflozin are both substrates of P-gp and BCRP, and canagliflozin is a weak inhibitor of P-gp, and when combined, the two drugs may cause drug interactions through competition or inhibition of the drug transporters. Interestingly, despite the fact that sorafenib, like lenvatinib, is a substrate for P-gp and BCRP, when co-administrated, the canagliflozin had little effect on the blood concentration-time profile of sorafenib. In the next study, the affinity of sorafenib and lenvatinib with the transporter protein P-gp and BCRP can be examined using molecular docking to explore the specific mechanism [51,52,53]. In addition, lenvatinib and canagliflozin are both high protein binding drugs and may compete for the same binding sites, leading to changes in free drug concentrations and causing changes in pharmacokinetics. Thus, drug interactions between lenvatinib and canagliflozin may be the result of a combination of multiple links and factors.

## 4. Materials and Methods

### 4.1. Materials

Sorafenib (purity: 99.5%, ZZS-20-638-G3), sorafenib-d_3_ (purity: 99.9%, ZZS-20-X261-A1), ^2^H_5_-lenvatinib (purity: 99.5%, ZZS-20-624-A9), canagliflozin (purity: 98%, ZZS19092401), and ^2^H_4_-canagliflozin (purity: 98%, 21J167-D1) were purchased from Shanghai Zhen Zhun Biological Technology Co., Ltd. (Shanghai, China). Lenvatinib (purity: 98%, Q75191201) was kindly provided by Shijiazhuang Pharmaceutical Group (Shijiazhuang, China). High-performance liquid chromatography (HPLC)-grade ammonium acetate, formic acid, acetonitrile, methyl tert-butyl ether, and ethyl acetate were obtained from Fisher Scientific Ltd. (Pittsburgh, PA, USA). Ultrapure water was acquired from Wahaha Group Co., Ltd. (Hangzhou, China).

### 4.2. Animals

Healthy male Sprague Dawley rats (body weight: 220–250 g) were purchased from Beijing Weitong Lihua Experimental Animal Technology Co., Ltd. (Beijing, China). The animals were kept in a controlled environment with a humidity of 40–60%, a temperature of 23–27 °C, a 12-h light/12-h dark cycle, and they were provided food and water ad libitum. All animal manipulations and experimental protocols were approved by the Ethics Committee of Hebei General Hospital (No. 2022011).

### 4.3. Preparation of Calibration Standards and Quality Control (QC) Samples

The stock solutions were prepared in dimethyl sulfoxide (DMSO), and the concentrations were 1 mg/mL for sorafenib, ^2^H_5_-lenvatinib, canagliflozin, and ^2^H_4_-canagliflozin; 2 mg/mL for lenvatinib; and 1.43 mg/mL for sorafenib-d_3_. A series of mixed working solutions containing sorafenib, lenvatinib, or canagliflozin were prepared by diluting with 50% acetonitrile–water and mixing. The stock solutions for sorafenib-d_3_, ^2^H_5_-lenvatinib, and ^2^H_4_-canagliflozin were prepared in the same manner with 50% acetonitrile–water, and the final concentrations of the mixture internal standard (IS) working solutions were 500, 500, and 2000 ng/mL, respectively. A 5 μL aliquot of the mixed working solution was added to 45 μL of blank plasma to prepare the calibration standards. The plasma standard points of the calibration curve were at the concentrations of 5, 15, 50, 200, 800, 2000, 4000, and 5000 ng/mL for sorafenib; 0.2, 1, 2, 10, 100, 200, 400, and 1000 ng/mL for lenvatinib; and 5, 20, 100, 500, 1000, 1500, 2000, and 3000 ng/mL for canagliflozin. The low, medium, and high QCs of sorafenib were 10, 1500, and 3750 ng/mL, respectively; the low, medium, and high QCs of lenvatinib were 0.5, 150, and 750 ng/mL, respectively; and the low, medium, and high QCs of canagliflozin were 10, 800, and 2250 ng/mL, respectively.

### 4.4. Plasma Sample Preparation

First, 50 μL of the plasma sample was added to 5 μL of the mixture IS working solution and 500 μL of the extraction reagent (methyl tert-butyl ether and ethyl acetate, 1:4) vortex-mixed for 3 min and then centrifuged at 12,000 rpm for 10 min. Next, 400 μL of supernatant was transferred into a new centrifuge tube, which was blown dry under a stream of nitrogen, and the residue was re-solubilized with 150 μL of 50% acetonitrile–water.

### 4.5. UPLC–MS/MS Conditions

The concentrations of analytes were determined by UPLC–MS/MS, which consisted of an LC-30A ultra-performance liquid chromatography (Shimadzu, Japan), and Sciex Triple Quad 5500 tandem triple quadrupole mass spectrometer (Applied Biosystems Sciex, Framingham, MA, USA). The XSelect HSS T3 column (2.1 mm × 100 mm, 2.5 μm, Waters, Milford, MA, USA) was used for chromatographic separation. Phase A (0.1% formic acid in ultrapure water containing 5 mM ammonium acetate) and phase B (acetonitrile) were used as the mobile phases for gradient elution at 0.35 mL/min. The following gradient conditions for phase B were used: 0–1 min, 60% B; 1–2 min, 60–90% B; 2–4 min, 90% B; 4.0–4.1 min, 90–60% B; and 4.1–5.1 min, 60% B. The positive ion mode with multi-reaction detection was used, and the multiple reaction monitoring transitions of the analytes were as follows: *m*/*z* 465.2→270.2 for sorafenib, 468.2→255.4 for sorafenib-d_3_, *m*/*z* 427.0→370.0 for lenvatinib, 432.1→370.0 for ^2^H_5_-lenvatinib, 462.1→191.3 for canagliflozin, and 466.1→195.3 for ^2^H_4_-canagliflozin.

### 4.6. Method Validation

The method for the simultaneous determination of sorafenib, lenvatinib, and canagliflozin was fully validated according to USA Food and Drug Administration guidelines and Chinese Pharmacopoeia. The selectivity of the method was assessed by comparing the chromatograms of six independent blank rat plasma samples, simulated plasma samples in which blank rat plasma samples were spiked with working solution at a lower limit of quantitation (LLOQ) and IS, and actual plasma samples collected from rats. The linearity of sorafenib, lenvatinib, and canagliflozin was evaluated using weighted least squares with the nominal concentration as the horizontal coordinate and the peak area ratio of the analyte to the IS as the vertical coordinate at concentration ranges of 5–5000 ng/mL, 0.2–1000 ng/mL, and 5–3000 ng/mL, respectively. Different concentrations of the QC and LLOQ samples, with six replicates at each concentration, were used to evaluate precision and accuracy, and inter-batch precision and accuracy were evaluated by measurements for three consecutive days. The precision was expressed as the coefficient of variation (CV%), and the accuracy was expressed as the relative error (RE%). The extraction recovery was evaluated by comparing the peak areas of blank plasma spiked with working solution pre-extraction and blank plasma spiked with working solution post-extraction. The matrix effect was evaluated by the ratio of the peak area of the analyte in the plasma to the peak area of the analyte in solution at three concentrations of the QC samples. The stability of sorafenib, lenvatinib, and canagliflozin in rat plasma was estimated under different storage and handling conditions. Three concentrations of the QC samples, with six replicates at each concentration level, were used to examine the stability of the three analytes at room temperature for 4 h, in the autosampler for 12 h, at −80 °C for 30 days, and freeze–thaw three times.

### 4.7. Pharmacokinetic and Drug–Drug Interaction Study

Before starting this experiment, the rats were fasted for 12 h, but were allowed to drink water freely. The experimental animals were randomly divided into five groups (n = 6 per group). Sorafenib gavage solution was prepared by 0.5% sodium carboxymethyl cellulose (CMC-Na) containing 5% DMSO, while lenvatinib and canagliflozin gavage solutions were prepared directly with 0.5% CMC-Na. Groups I–III were control groups, and rats in these groups were gavaged with sorafenib at 100 mg/kg (I_SOR_), lenvatinib at 1.2 mg/kg (II_LEN_), and canagliflozin at 10 mg/kg (III_CA_), respectively. Rats in Group IV were gavaged with sorafenib at 100 mg/kg and canagliflozin at 10 mg/kg (IV_SOR+CA_), and rats in Group V were gavaged with lenvatinib at 1.2 mg/kg and canagliflozin at 10 mg/kg (V_LEN__+CA_). Approximately 0.25 mL of blood was collected from the orbital venous plexus of rats in pre-heparinized centrifuge tubes. The blood collection time points were as follows: 0, 0.5, 1, 1.5, 2, 3, 4, 5, 6, 7, 8, 12, 24, 48, 72, and 96 h for sorafenib; 0, 0.25, 0.5, 1, 1.5, 2, 3, 4, 5, 6, 7, 8, 12, 24, 48, 72, and 96 h for lenvatinib; and 0, 0.25, 0.5, 1, 1.5, 2, 3, 4, 5, 6, 7, 8, 12, 24, 48, and 72 h for canagliflozin. Blood samples were centrifuged at 3500 rpm for 10 min, and the plasma was transferred to new centrifuge tubes and then stored at −80 °C.

The C_max_ and T_max_ (time to maximum plasma concentration) were read directly from the drug concentrations. The other pharmacokinetic parameters were analyzed using a non-compartmental analysis with Phoenix WinNonLin 8.2 software (Pharsight, Mountain View, Certara, CA, USA). The other pharmacokinetic parameters were as follows: AUC_last_, area under the plasma concentration–time curve from time zero to last time point; AUC_inf_, area under the plasma concentration–time curve from time zero to infinity; t_1/2_, time required for a 50% decrease in the plasma drug concentration; CL_z__/F_, volume of plasma that is cleared of the drug per unit time; and V_z__/F_, apparent volume of distribution.

### 4.8. Statistical Analysis

All pharmacokinetic results were expressed as the mean ± standard deviation (SD), and the T_max_ was expressed as the median (range). SPSS 25.0 software was used for statistical analysis, and *p* < 0.05 was considered a statistically significant difference. Shapiro–Wilk test was used to assess the normality of the pharmacokinetic parameters, and Student’s *t*-test or Mann–Whitney U test was used based on the results of the normality test.

## 5. Conclusions

This study established an UPLC-MS/MS method for the simultaneous determination of sorafenib, lenvatinib, and canagliflozin in rat plasma. The method was successfully applied to pharmacokinetic drug interaction studies between canagliflozin and sorafenib or lenvatinib. The results indicated that there were drug interactions between sorafenib and canagliflozin, and the simultaneous administration of sorafenib can significantly increase the in vivo systemic exposure of canagliflozin. Although drug interactions exist between lenvatinib and canagliflozin, but the results are different from the drug interaction between sorafenib and canagliflozin. The presence of lenvatinib led to a decrease in the V_z/F_ and CL_z/F_ of canagliflozin, while the presence of canagliflozin led to a significant increase in the in vivo systemic exposure of lenvatinib, and a decrease in the V_z/F_ and CL_z/F_ of lenvatinib. Considering the increased risk of HCC in patients with T2DM and the fact that SGLT2 is expressed on certain cancer cells including HCC and canagliflozin has been reported to inhibit the growth of hepatoma cells, it is expected that the opportunity for combined use of canagliflozin and sorafenib/lenvatinib in clinical practice will continue to increase in the future. This study may provide an important reference for the use of drugs in patients with HCC with T2DM in clinical practice.

## Figures and Tables

**Table 1:** Precision and accuracy of sorafenib, lenvatinib, and canagliflozin in rat plasma.

Analytes	Concentration (ng/mL)	Intra-Batch (n = 6)			Inter-Batch (n = 18)		
		Mean ± SD	CV (%)	RE (%)	Mean ± SD	CV (%)	RE (%)
Sorafenib	5	5.32 ± 0.29	5.42	6.30	5.48 ± 0.26	4.72	9.57
	10	10.08 ± 0.59	5.86	0.80	10.60 ± 0.60	5.70	6.04
	1500	1481.67 ± 84.72	5.72	−1.22	1471.67 ± 97.03	6.59	−1.89
	3750	3900.00 ± 264.20	6.77	4.00	3855.59 ± 258.05	6.69	2.81
Lenvatinib	0.2	0.21 ± 0.02	9.54	6.08	0.22 ± 0.02	8.24	8.03
	0.5	0.52 ± 0.02	4.76	3.20	0.51 ± 0.04	7.51	2.43
	150	147.67 ± 5.92	4.01	−1.56	149.33 ± 10.13	6.78	−0.44
	750	715.83 ± 30.51	4.26	−4.56	738.78 ± 43.23	5.85	−1.50
Canagliflozin	5	5.49 ± 0.21	3.75	9.73	5.26 ± 0.42	7.93	5.10
	10	10.36 ± 0.46	4.43	3.55	10.31 ± 0.59	5.70	3.06
	800	782.83 ± 42.24	5.40	−2.15	787.28 ± 66.44	8.44	−1.59
	2250	2155.00 ± 103.10	4.78	−4.22	2200.56 ± 119.63	5.44	−2.20

**Table 2:** Matrix effect and extraction recovery of sorafenib, lenvatinib, and canagliflozin in rat plasma (n = 6).

Analytes	Concentration (ng/mL)	Matrix Effect		Extraction Recovery	
		Mean ± SD (%)	CV (%)	Mean ± SD (%)	CV (%)
Sorafenib	10	105.44 ±4.81	4.57	97.55 ± 4.03	4.13
	1500	99.57 ± 7.01	7.04	100.18 ± 3.67	3.66
	3750	99.98 ± 5.08	5.08	100.59 ± 6.84	6.80
Lenvatinib	0.5	103.74 ± 11.20	10.80	89.97 ± 8.48	9.43
	150	95.67 ± 10.44	10.91	100.08 ± 9.47	9.47
	800	96.65 ± 2.17	2.25	99.40 ± 9.27	9.33
Canagliflozin	10	101.05 ± 9.13	9.03	97.07 ± 9.49	9.78
	800	95.82 ± 7.21	7.52	100.25 ± 7.81	7.79
	2250	103.16 ± 3.72	3.61	96.85 ± 6.22	6.42

**Table 3:** Stability of sorafenib, lenvatinib, and canagliflozin in rat plasma under various storage conditions (n = 6).

Analytes	Concentration (ng/mL)	Bench-Top ^a^	Autosampler ^b^	Freeze-Thaw ^c^	Long-Term ^d^
Sorafenib	10	10.97 ± 0.29	11.13 ± 0.24	10.55 ± 0.43	10.51 ± 0.43
	1500	1551.67 ± 84.00	1540.00 ± 95.71	1531.67 ± 117.88	1565.00 ± 123.25
	3750	3948.33 ± 99.88	3961.67 ± 102.26	3698.33 ± 72.78	3583.33 ± 67.43
Lenvatinib	0.5	0.54 ± 0.03	0.53 ± 0.04	0.53 ± 0.03	0.47 ± 0.03
	150	156.67 ± 7.42	162.67 ± 7.42	157.33 ± 11.02	157.00 ± 12.63
	800	794.00 ± 18.24	819.50 ± 30.36	725.83 ± 18.06	714.00 ± 24.44
Canagliflozin	10	10.87 ± 0.52	11.13 ± 0.23	9.60 ± 0.45	10.02 ± 0.71
	800	849.83 ± 44.57	847.67 ± 52.93	854.50 ± 48.73	830.00 ± 42.37
	2250	2330.00 ± 80.00	2350.00 ± 72.94	2183.33 ± 82.62	2170.00 ± 59.67

^a^ Room temperature for 4 h; ^b^ autosampler for 12 h; ^c^ freeze–thaw stability for three times; ^d^ −80 °C for 30 days.

**Table 4:** Pharmacokinetic parameters of sorafenib in rat plasma after oral administration of single dose sorafenib (100 mg/kg) and combined with canagliflozin (10 mg/kg); n = 6 for each group.

Parameters (Unit)	I_SOR_	IV_SOR+CA_	*p*-Value
t_1/2z_ (h)	12.30 ± 2.23	10.47 ± 0.94	0.109
C_max_ (ng/mL)	1916 ± 298	2195 ± 535	0.292
AUC_last_ (h·ng/mL)	39,970 ± 14,538	41,158 ± 9197	0.869
AUC_inf_ (h·ng/mL)	40,273 ± 14,696	41,290 ± 9225	0.889
T_max_ (h)	8.00 (7.00–8.00)	6.50 (5.50–7.25)	0.065
CL_z/F_ (L/h/kg)	2.86 ± 1.30	2.52 ± 0.53	0.569
V_z/F_ (L/kg)	49.00 ± 18.83	38.30 ± 10.12	0.248

I_SOR_, sorafenib (100 mg/kg); IV_SOR+CA_, sorafenib (100 mg/kg) oral co-administered with canagliflozin (10 mg/kg). T_max_ was presented as median (range), and the other pharmacokinetic parameters are expressed as the mean ± standard deviation. Compared with sorafenib alone, indicating statistically significant difference.

**Table 5:** Pharmacokinetic parameters of canagliflozin in rat plasma after oral administration of single dose canagliflozin (10 mg/kg) and combined with sorafenib (100 mg/kg) and lenvatinib (1.2 mg/kg); n = 6 for each group.

Parameters (Unit)	III_CA_	IV_SOR+CA_	V_LEN__+CA_	III_CA_ vs. IV_SOR+CA_ *p*-Value	III_CA_ vs. V_LEN__+CA_ *p*-Value
t_1/2z_ (h)	8.84 ± 0.35	8.56 ± 0.61	10.12 ± 3.59	0.347	0.394
C_max_ (ng/mL)	3096 ± 776	4103 ± 335	3008 ± 484	0.041 *	0.818
AUC_last_ (h·ng/mL)	48,875 ± 7446	67,320 ± 3637	67,970 ± 18,572	0.002 **	0.054
AUC_inf_ (h·ng/mL)	49,103 ± 7508	67,554 ± 3597	68,654 ± 19,418	0.002 **	0.058
T_max_ (h)	3.50 (3.00–6.25)	5.50 (3.50–6.00)	4.00 (3.74–4.50)	0.699	0.818
CL_z/F_ (L/h/kg)	0.21 ± 0.03	0.15 ± 0.01	0.15 ± 0.04	0.002 **	0.029 *
V_z/F_ (L/kg)	2.65 ± 0.39	1.84 ± 0.22	2.12 ± 0.32	0.001 **	0.030 *

III_CA_, canagliflozin (10 mg/kg); IV_SOR+CA_, canagliflozin (10 mg/kg) oral co-administered with sorafenib (100 mg/kg); V_LEN+CA_, canagliflozin (10 mg/kg) oral co-administered with lenvatinib (1.2 mg/kg). T_max_ was presented as median (range), and the other pharmacokinetic parameters are expressed as the mean ± standard deviation. * *p* < 0.05, ** *p* < 0.01, compared with canagliflozin alone, indicating statistically significant difference.

**Table 6:** Pharmacokinetic parameters of lenvatinib in rat plasma after oral administration of single dose lenvatinib (1.2 mg/kg) and combined with canagliflozin (10 mg/kg); n = 6 for each group.

Parameters (Unit)	II_LEN_	V_LEN__+CA_	*p*-Value
t_1/2z_ (h)	10.48 ± 4.53	6.49 ± 1.04	0.004 **
C_max_ (ng/mL)	721 ± 144	985 ± 162	0.014 *
AUC_last_ (h·ng/mL)	5640 ± 1292	7281 ± 1167	0.044 *
AUCinf (h·ng/mL)	5653 ± 1299	7285 ± 1167	0.045 *
T_max_ (h)	1.75 (1.38–2.75)	1.50 (0.88–1.63)	0.240
CL_z/F_ (L/h/kg)	0.22 ± 0.05	0.17 ± 0.03	0.047 *
V_z/F_ (L/kg)	3.31 ± 1.51	1.56 ± 0.29	0.004 **

II_LEN_, lenvatinib (1.2 mg/kg); V_LEN+CA_, lenvatinib (1.2 mg/kg) oral co-administered with canagliflozin (10 mg/kg). T_max_ was presented as median (range), and the other pharmacokinetic parameters are expressed as the mean ± standard deviation. * *p* < 0.05, ** *p* < 0.01, compared with lenvatinib alone, indicating statistically significant difference.

## Data Availability

Not applicable.

## Sample Availability

Samples of the compounds are not available from the authors.
